# Development of an LC-MS/MS Method for Non-Invasive Biomonitoring of Neonicotinoid and Systemic Herbicide Pesticide Residues in Bat Hair

**DOI:** 10.3390/toxics10020073

**Published:** 2022-02-05

**Authors:** Sarah E. Hooper, Sybill K. Amelon, Chung-Ho Lin

**Affiliations:** 1 Department of Biomedical Sciences, Ross University School of Veterinary Medicine, P.O. Box 334, Basseterre, St. Kitts KN0101, West Indies; 2 Department of Veterinary Pathobiology, College of Veterinary Medicine, University of Missouri, 201 Connaway Hall, Columbia, MO 65211, USA; 3 USDA Forest Service Northern Research Station, 202 ABNR Building, 1111 Rollins St., Columbia, MO 65211, USA; sybill.amelon@usda.gov; 4 Center for Agroforestry, University of Missouri, 302 ABNR Building, 1111 Rollins St., Columbia, MO 65211, USA; linchu@missouri.edu

**Keywords:** bats, Chiroptera, ecotoxicology, glyphosate, neonicotinoid, non-invasive methods, pesticide residues, pesticide exposure

## Abstract

With over a quarter of the world’s bats species facing extinction, there is a need for ecotoxicological studies to assess if acute and sublethal exposure to newer pesticides such as neonicotinoids and carbonates contribute to population declines. Pesticide exposure studies in bats have been limited to terminal sampling methods, therefore we developed a non-invasive liquid chromatography-tandem mass spectrometry (LC-MS/MS) method utilizing hair trimmings. The hair of big brown bats (*Eptesicus fuscus*) was collected and pooled by county to assess the best extraction solvent and solid-phase-extraction (SPE) clean-up cartridges. Using the best performing extraction solvent, methanol, and the best performing SPE cartridge, Chromabond HR-X, we developed an optimized multiple reaction monitoring (MRM) LC-MS/MS method for simultaneous determination of 3 neonicotinoids, clothianidin, imidacloprid, and thiamethoxam; 1 carbonate, carbaryl; and 4 systemic herbicides, 2,4-D, atrazine, dicamba, and glyphosate. The optimized protocol yielded the detection of 3–8 of the compounds in the county-level bat hair pools. 2,4-D, glyphosate, and imidacloprid were found in all samples with two of the county-level hair samples having glyphosate concentrations of over 3500 pg/mg of hair. This approach has great potential to facilitate non-terminal ecotoxicological studies assessing the effects of subacute (chronic) pesticide exposure in threatened and endangered bat species and other species experiencing population declines.

## 1. Introduction

Over 25% of the world’s nearly 1400 bat species face extinction [1,2,3] and over half of Chiropteran species assessed by the International Union for Conservation of Nature (IUCN) have decreasing populations or an unknown population status [2]. This decrease in bat abundance and diversity is alarming because bats play a vital role in ecosystem health [4] by providing essential ecosystem functions such as arthropod suppression, seed dispersal, and pollination [5,6]. Possible reasons provided for these declines frequently include habitat loss, white-nose syndrome, wind energy, and most recently, climate change [3,7]. Surprisingly, pollutants, including pesticides, are rarely cited as contributing to population declines.

The few bat-focused ecotoxicological studies of pollutants refer to heavy metals and the measurement of organochlorine residue concentrations in tissues [7,8,9]. Organochlorines were widely utilized in the 1940s–1960s to combat malaria and other insect-borne diseases in urban populations and insect control in crop and livestock production and household pest control [10]. Accompanying the widespread use of these organochlorines were severe adverse environmental effects, largely ignored until Rachel Carson published her book “Silent Spring” [11]. Carson highlighted the widespread, severe non-target effects of DDT and other organochlorines on wildlife, focusing on embryotoxicity and birds’ eggshell thinning causing severe avian population declines [12].

With increased public awareness and subsequent outrage, DDT was outlawed in the US in 1972 with Canada and European countries following suit [10,11]. Due to their persistence in the environment, several studies have reported measuring DDT, other organochlorines, and metabolites of these compounds in bat tissues, however, since the banning of their use, the concentration of these contaminants in bat tissue have continually declined since initial tissue concentrations were reported in the 1970s and 1980s [7].

Other pesticides were introduced following the banning of organochlorines, including pyrethroids, neonicotinoids, and carbamates [13]. In 2014, worldwide use of these newer pesticides was estimated to be two million tons with herbicides composing 47.5% of usage and insecticides comprising 17.5% of use [14]. Six years later in 2020, worldwide pesticide use is expected to nearly double to an estimated 3.5 million tons [14]. With the increasing use of pesticides, some non-organochlorines such as glyphosate are now ubiquitous in our environment [15], indicating a need for ecotoxicological studies to assess these new pesticides and their potential risks to chiropteran species [7,9].

The few chiropteran ecotoxicological studies on organochlorines have relied on whole carcass or organ analysis [8,9]. Sharp declines worldwide of Chiropteran populations and rising numbers of threatened and endangered bat species [1,2,3], suggest development of new non-lethal methods for ecotoxicological studies, as euthanizing large numbers of bats on the landscape is not a viable nor a sustainable option.

Several studies report using hair to assess chronic metal exposure and accumulation in wildlife species [16,17]. Hair is routinely clipped from the interscapular region during placement of very high frequency (VHF) telemetry transmitters [18]. Our objective is to determine if and develop a highly sensitive and specific liquid chromatography (LC) tandem mass spectrometry (MS/MS) method to utilize the discarded hair from telemetry placement to quantify contaminant exposure in bats.

## 2. Materials and Methods

### 2.1. Reference Substances, Chemicals, Solvents

All analytical standards (2,4-dichlorophenoxyacetic acid, atrazine, 3,6-Dichloro-2-methoxybenzoic acid, glyphosate, carbaryl, clothianidin, imidacloprid, thiamethoxam, imidacloprid-d4, 3,6-Dichloro-2-methoxybenzoic acid-d4) were Supelco PESTANAL^®^ certified analytical pesticide standards purchased from Sigma–Aldrich (Saint Louis, MO, USA). All reagents were UPLC-MS grade (Optima™ LC/MS, Thermo Fisher Scientific, Waltham, MA, USA) except formic acid which was HPLC grade (Sigma-Aldrich, St. Louis, MO, USA). Ultrapure MilliQ water (Millipore, Bedford, MA, USA) was used to prepare mobile phases and extraction processes.

### 2.2. County Pesticide Application Estimate—Selection of Pesticides

Low and high county-level estimated agricultural pesticide use was obtained from the United States Geologic Survey (USGS) Pesticide National Synthesis Project from the counties where bats were collected. The United States Department of Agriculture (USDA) 2017 Census of Agriculture “land in farms” was used to determine the amount applied to farmland for four target herbicides, 2,4-D, atrazine, dicamba, and glyphosate; and four target pesticides, imidacloprid, clothianidin, thiamethoxam, and carbaryl using calculations based on avoided use economic models [19] to report estimates in kilograms/land in farms in kilometers.

### 2.3. Sample Collection

Whole carcasses of rabies negative big brown bats (*Eptesicus fuscus*) were obtained from the Missouri State Public Health Laboratory Virology Unit. The hair of each big brown bat was washed with ultrapure (MilliQ) water for one minute and subsequently washed for thirty seconds with isopropanol to remove external contamination according to the Society of Hair Testing (SoHT) Guidelines for Drug Testing in Hair [20]. The isopropyl and ultrapure water can be saved for analysis if external pesticide contamination desires to be measured. Intrascapular hair was clipped as if in preparation for a very high frequency (VHF) transmitter placement to determine if the amount obtained was adequate for analysis of pesticide residues (Figure S1). Hair was weighed to determine the sample weight for each replicate sample. To allow the testing, multiple extraction solvents and clean-up solid-phase extraction (SPE) cartridges for one target herbicide and one target pesticide, the hair of three big browns was cleaned, clipped, and pooled into a single hair sample which was then used to test each method concurrently.

### 2.4. Hair Sample Preparation

Three replicates of fifteen-milligram aliquots were used to assess recovery rates using three extraction solvent protocols and three solid-phase extraction (SPE) columns reported to successfully extract similar pesticide compounds from biological material. Approximately fifteen milligrams of hair per sample replicate were pulverized with a mortar and pestle. The pulverized hair was weighed to the nearest hundreds of a gram and transferred to a glass 13 × 100 mm tube. Three mL of one of the following was added to the 13 × 100 mm tube: methanol, acetonitrile, or a 1:1 solution of acetonitrile: Millipore water. Ten nanograms of a working internal standard solution (0.1 ppm of imicloprid-d4 and dicamba-d3) were added subsequently. Each sample was sonicated for 30 min in a 40 °C water bath and incubated overnight on a rotating plate set at 160 rpm. Samples were evaporated under streaming nitrogen at room temperature and resuspended in 1 mL of 2% methanol for the methanol extractions or 1 mL of 10% acetonitrile for the acetonitrile and acetonitrile-water extractions. 

### 2.5. Hair Sample Cleanup

Hair extracts were subsequently cleaned by using one of the following solid-phase extraction (SPE) cartridges: Chromabond cartridge packaged with 30 mg HR-X sorbent (1 mL) (Macherey-Nagel, Bethlehem, PA, USA); HyperSep cartridge packaged with 1000 mg Aminopropyl sorbent (6 mL) (Thermo Scientific); or a Waters Oasis cartridge packed with 60 mg of Hydrophilic-Lipophilic-Balance (HLB) 60 μm sorbent (3 mL) with a 12 port, SPE vacuum manifold (Restex, Bellefonte, PA, USA). The Chromabond HR-X and Oasis HLB columns were conditioned with 6 mL of acetonitrile followed by 6 mL of ultrapure water. The HyperSep column was conditioned with 6 mL of acetonitrile, 6 mL of dichloromethane, and then 6 mL of acetonitrile [21].

After conditioning, the resuspended extract was loaded at a flow rate of 0.5–1.0 mL/min and subsequently dried for 5 min. Samples were eluted with 6 mL of 100% methanol followed by 6 mL of 100% acetonitrile. The extracts were evaporated under streaming nitrogen at room temperature and resuspended in 500 µL of 100% acetonitrile. Each sample was filtered with a 13 mm, 0.2 µL PTFE Acrodisc Syringe Filter (Waters Corporation, Milford, MA, USA) before injection.

### 2.6. Optimization of MRM Transition Parameters

One precursor and the product ion(s) for each pesticide of interest were selected by first running the single MS full scan mode on a Waters 2695 high-performance liquid chromatography system coupled with a UV detector (Waters 996 photodiode array detector) and a Waters Acquity TQ triple quadrupole mass spectrometer (MS/MS) (Waters TQ Detector, Acquity ultra-performance LC). Subsequently, the product ion scan mode was performed. Within the Waters Empower 3 Chromatogaphy software, the AutoTune was performed on each individual analyte using the obtained precursor ion and the product ions. The MRM experimental optimal parameters were selected from the generated report and the precursor scan was used to determine retention time.

### 2.7. Pesticide Quantification by Liquid Chromatography Tandem Mass Spectrometry (LC-MS/MS)

All pesticides were simultaneously measured using a Waters 2695 high-performance liquid chromatography system coupled with a UV detector (Waters 996 photodiode array detector) and a Waters Acquity TQ triple quadrupole mass spectrometer (MS/MS) (Waters TQ Detector, Acquity ultra-performance LC), controlled by the Waters Empower 3 Chromatography software. A reverse-phase C_18_ HPLC column (Kinetex 2.6 µm C_18_ 100 Å, LC column 100 mm × 4.6 mm; Phenomenex, Torrance, CA, USA) heated to 40 °C was used for chromatographic separation of target pesticides. Each run was 15 min using a solvent flow rate of 0.5 mL min^−1^ with the following gradient: 0 min, 2% A (100% acetonitrile with 0.1% (*v/v*) formic acid), 98% B (MilliQ water with 0.1% (*v/v*) formic acid); 7.27 min, 80% A, 20% B; 7.37 min, 98% A, 2% B; 10 min, 2% A, 98% B. For each target compound and internal standard, the full spectrum of the protonated [M + H]^+^ or deprotonated [M − H]^−^ molecular (precursor) ion was generated. Subsequently, the full spectrum of the product ions was generated with the predominant fragmented ion selected for quantification. The ion source in the MS/MS system was electron spray ionization (ESI) with the target compound dictating whether positive or negative ion mode was used with the capillary voltage of 1.5 kV. The ionization sources were programmed at 150 °C and the desolvation temperature was programmed at 450 °C. The MS/MS system was operated in the multiple reaction monitoring (MRM) mode with the cone voltage and collision energy based upon the optimization parameters for each compound. All pesticide concentrations were reported as picograms per milligram of hair.

### 2.8. Limit of Detection and Limit of Quantification

The limit of detection (LOD) was calculated using the following equation:

LOD=3.3×σ÷S

where σ equals the standard deviation of the response and S equals the slope of the calibration curve for the analyte [22]. The limit of quantification (LOQ) which corresponds to the lowest concentration that can be measured with acceptable accuracy and precision was calculated using the following equation:

LOQ=10×σ÷S

### 2.9. Recovery Assay

Blank hair samples were spiked in triplicate at three concentration levels, 1 ppb, 10 ppb, and 100 ppb. Samples were prepared and pesticides quantified as described above. The mean recoveries were calculated as follows:

$$\mathrm{Mean}\;\mathrm{recovery}\;\mathrm{percent}=\frac{\mathrm{spiked}\;\mathrm{sample}-\mathrm{blank}\;\mathrm{sample}}{\mathrm{measured}\;\mathrm{spiking}\;\mathrm{concentration}}\times 100\%$$

Inter-assay coefficient of variation was calculated as follows:

$$\mathrm{Inter}-\mathrm{assay}\;\mathrm{coefficient}\;\mathrm{of}\;\mathrm{variation}=\frac{\mathrm{pooled}\;\mathrm{standard}\;\mathrm{deviation}}{\mathrm{overall}\;\mathrm{mean}\;\mathrm{of}\;\mathrm{replicate}\;\mathrm{samples}}\times 100\%$$

### 2.10. Matrix Effect

The matrix effect was analyzed using a post-extraction spike matrix comparison as outlined by Matuszewski et al. 2003 [23] and Panuwet et al. 2016 [24]. Three aliquots of fifteen milligrams of blank hair were extracted. Each extract was spiked with 1 ppb, 100 ppb, and 1000 ppb. The absolute matrix effects were calculated using the following equation:

$${\mathrm{ME}}_{\mathrm{ionization}}=\frac{{\mathrm{Analyte}\;\mathrm{signal}}_{\mathrm{post}\;\mathrm{extraction}\;\mathrm{spiked}\;\mathrm{matrix}}}{{\mathrm{Analyte}\;\mathrm{signal}}_{\mathrm{solvent}}}\times 100\%$$

An ME_ionization_ value greater than 100% indicates ionization enhancement and a value less than 100% indicates ionization suppression.

## 3. Results

### 3.1. Pesticide Agricultural Application in Missouri

The hair pools were from bats from Audrain (state and county FIP code 29007), Platte (state and county FIP code 29165), and St. Louis (state and county FIP code 29189) counties in Missouri. Table 1 shows the estimated kg per square km applied to agricultural land within the target counties and the national averages. National averages and county-level averages for Thiamethoxam, estimates were based upon the visualizations available on the Pesticide National Synthesis Project website as the data tables in the repository were incomplete (e.g., thiamethoxam use is present in Missouri based upon the visualizations, but the data tables only listed pesticides starting with the letters A to I).

### 3.2. Extraction and Clean-Up Assessment

The best performing extraction protocol using pure methanol and the best performing SPE column, Chromabond HR-X, resulted in 70.8% to 121% recovery rates for the target pesticides (Table 2). The poorer performing acetonitrile and 1:1 acetonitrile-Millipore water and Oasis HLB columns resulted in 7.9% to 142.1% recovery rates and the Hypersep aminopropyl column resulted in 10.2% to 172.0%. Each pesticide’s calibration curves were linear 0.1 ppb to 1000 ppb with *R*^2^ values of 0.99, except glyphosate with a logarithmic calibration curve. If standards over 1000 ppb were used, the calibration curves became non-linear and best-fit trend line was polynomial. All CVs were below the acceptable maximum of 15%.

### 3.3. Absolute Matrix Effects

The absolute matrix effects were only able to be calculated for the blank hair spiked with the 100 ppb and 1000 ppb pesticide standards as 1 ppb blank hair samples were below the LOQ for all pesticides except carbaryl. The negative ESI mode analytes, 3,6-Dichloro-2-methoxybenzoic acid (dicamba) and 2,4-dichlorophenoxyacetic acid (2,4-D) exhibited the most ionization suppression within the hair matrix with ME_ionization_ calculated to be 69.9% and 83.6% at 100 ppb (Table 2) with similar ME_ionization_ at 1000 ppb (data not shown). The neonicotinoid pesticides showed minimal matrix effects, and atrazine was slightly enhanced at 100 ppb and 1000 ppb.

### 3.4. Determination of Pesticides in Bat Hair

The three county-level hair pools analyzed using the optimized parameters are reported in Table 3. The selected transitions for each target pesticide compound showed good specificity and there were no interfering peaks observed. 2,4-dichlorophenoxyacetic acid (2,4-D), glyphosate, and imidacloprid were found in all hair samples. One county-level hair sample had all eight target pesticides detected whereas one hair sample had only four target pesticides detected (Table 3). The pesticide concentrations were highly variable, with the highest measured concentration was glyphosate at 4505.2 pg/mg and imidacloprid the lowest concentration with 10.6 pg/mg.

## 4. Discussion

Despite the US’s 1970s ban on DDT and other organochlorides [10,11], these compounds have remained in the environment due to their stability and resistance to degradation. Localized, “hot spot” locations, where heavy use of these persistent organic pollutants occurred, continue to be a threat to wildlife [8,11]. While this is well documented, the focus of chiropteran ecotoxicological studies continues to remain limited to these banned compounds [8,9,25].

Over the past 50 years, new pesticides have been introduced to replace organochlorides, leading to some non-organochlorine pesticides, becoming ubiquitous in our environment [15]. Risks imposed by popularization of these ubiquitous pesticides require chiropteran focused ecotoxicological studies [7,26]. Non-lethal methods for assessing acute and sublethal pesticide exposure in bat species are needed since existing terminal methods are not viable for the rapidly declining US bat populations [26,27]. We addressed this critical need by developing a non-lethal LC-MS/MS-based method for non-invasively quantifying pesticides in bat hair.

Hair, blood, feces, breastmilk and urine have been routinely used in humans for biomonitoring of pesticides [28]. Of these, hair collection is the least invasive for bats. Hair is simple to collect, and field technicians can be easily trained in the sampling technique. Hair is a stable matrix and therefore useful for assessing sublethal and chronic effects of environmental contaminants [29], whereas guano likely would only yield acute exposure to pesticides, such as exposure the evening the bat was captured or the prior evening [30]. Additionally, collecting feces from threatened and endangered bat species may require bats to be held longer than 30 min, the commonly specified time limit under the US Fish and Wildlife Service (USFWS) Endangered Species Recovery Permits [31]

Hair is an appealing option as it represents incorporation of the compounds systemically. Hair is routinely clipped for radio telemetry studies of bat life history and ecology [18] or under contracts by clients to meet the legal requirements of environmental impact assessments for regulated development projects [32,33]. Hair for transmitter placement is routinely clipped from the dorsum [18] and disposed of as it has no further use. The methodology presented in this paper demonstrates how this hair is a valuable resource for assessing pesticide levels.

Being a stable matrix, the hair does not require special transport and storage requirements and can be easily stored in the field for an extended period of time [30]. The observed matrix effects were minimal for the neonicotinoids, carbaryl, and atrazine. Some ion suppression was notable for 2,4-dichlorophenoxy acetic acid (2,4-D) and 3,6-Dichloro-2-methoxy-benzoic acid (Dicamba), and glyphosate which may be partially resolved by injecting a smaller amount. Since the other analytes of interest were minimally suppressed or enhanced, then isotopically labeled internal standards for those three compounds may be the most effective way to account for matrix effects since matrix-matching calibration may not be an option (Panuwet et al., 2016).

The amount of hair typically removed for placement of a transmitter proved to be sufficient for measuring eight commonly used pesticides (Table 3). Hair pools were used rather than samples from individual bats to enable evaluation of different extraction solvents and SPE cartridges in triplicate and to allow optimization of protocols but would not be required for routine analysis. County-level results demonstrated the value of our method as this is the first report of newer, commonly used pesticides being incorporated into bat tissues. Based upon the estimated county-level pesticide application (Table 1), our results suggest that exposure to pesticides may be linked to environmental loads within land-use classes. Analysis of bat hair collected in radio-telemetry studies may be valuable to pinpoint the location of environmental exposure that can be paired with environmental sampling to determine if exposure is from food consumption, water consumption, or the roosting environment.

Our study supports the use of bat hair to bio-monitor pesticides in agricultural, urban, and other ecosystems as the estimates provided by USGS’s Pesticide National Synthesis Project (Table 1) only include pesticides applied to agricultural land, and not residential or urban areas. Due to only agriculture uses being monitored, pesticide use doesn’t represent all sources that may exist [34]. Beginning in 2015, the estimates no longer included certain types of pesticide applications such as seed coatings [34]. Our results confirm these estimates don’t completely assess the risk of pesticide exposure to bats as clothianidin was not reported in any county in Missouri post-2015. The neonicotinoid was detected in two of the samples and was the second-highest concentration of pesticide measured in the Audrain County hair pool (Table 3). Understanding the risk of exposure is the first step towards understanding which pesticides should be investigated in ecotoxicological studies.

Our results (Table 3) suggest bats encounter pesticides routinely used in seed coatings and those found in a number of residential consumer products [35]. Prior research has shown that less than 2% of pesticides put on the seed are incorporated into the plant as it grows [36] indicating many of these persistent, water-soluble pesticides have potentially long-lasting non-target effects [37]. Up to 90% of some crops such as corn are pesticide-coated seeds, yet only 65% of corn growers and 43% of spring wheat growers could provide the names of the seed treatment product on their crops which may help explain why seed-coated pesticides are routinely under-reported [38]. Our data support these pesticides are routinely under-reported due to finding high concentrations of clothianidin and thiamethoxam despite having the lowest estimated use (Table 1) or no reported use. The LC-MS/MS method presented here allows a non-terminal, temporal and spatial survey of the exposure risks to bats for three neonicotinoids which account for the majority of insecticides used in seed coatings [39].

While the half-life of neonicotinoids in soil can be up to 3.5 years [40], some of the target herbicides such as glyphosate have a relatively short half-life, with an estimated 7 to 60 days [41]. Despite the short half-life, glyphosate concentrations were found to be as high as 4505 pg/mg of hair (Table 3), the highest of any target pesticide. This could indicate that a short-half life may not reduce the risk of exposure as environmental loads may be extremely high [15,41].

## 5. Conclusions

Pesticides refer to a broad range of chemicals that are designed to control target organisms such as insects (insecticides), plants (herbicides), and other organisms (e.g., fungicides and algicides). While judicious use of pesticides can be beneficial, there is always a risk of non-target effects of pesticides.

With the increased use of newer pesticides such as neonicotinoids and carbamates [13,14], there have been calls to understand the ecotoxicology and quantification of acute and sublethal exposure to pesticides. The LC-MS/MS-based method we developed for quantifying contaminants in bat hair, addresses this major gap in research by bringing the molecular capabilities to analyze newer pesticides such as neonicotinoids. It additionally addresses the research need to use non-invasive samples when working with species of conservation concern. This method opens opportunities to better understand if contaminant exposures result in sublethal, acute, or chronic effects and whether synergistic effects of multiple threats are associated with continuing declines of bat populations.

## Figures and Tables

**Table 1:** USGS 2017 high and low approximate estimate ranges of annual agricultural pesticide use for Audrain, Platte, St Louis counties in Missouri. County-level estimates do not include estimates for seed treatment application of pesticides nor non-agricultural (consumer) products. * Estimates based upon USGS map of estimated use on agriculture land as data was not included in USGS county-level data tables.

Pesticide	Category	Class	Estimated Applied Amount (kg per km^2^)			Total National Estimated Use (million kgs)
			Audrain	Platte	St. Louis	
2,4-dichlorophenoxyacetic acid (2,4-D)	Herbicides	Systemic herbicide	7.7–30.4	167.5–168.1	25.2–27.8	19.5–20.4
Atrazine	Herbicides	Systemic herbicide	272.3	326.3–27.1	54.9	32.7–33.6
3,6-Dichloro-2-methoxybenzoic acid (Dicamba)	Herbicides	Selective herbicide	64.1–66.2	41.9–42.3	12.6–12.7	7.7–9.1
Glyphosate	Herbicides	Systemic herbicide	796.5–801.2	792.8–793.2	244.9–245.1	122.5–127.0
Carbaryl	Insecticides	Carbamates	0.2	0.4	0.08	0.3–0.7
Clothianidin	Insecticides	Neonicotinoid	No estimated use	No estimated use	No estimated use	0.05–0.09
Imidacloprid	Insecticides	Neonicotinoid	0–1.0	0.02	0.65	0.5–0.6
Thiamethoxam	Insecticides	Neonicotinoid	0–0.9 *	0.002–0.91 *	0.002–0.91 *	0.09 to 0.11

**Table 2:** HPLC-MS/MS optimized parameters including the quantifier and qualifier ion, limit of detection (LOD), and limit of quantification (LOQ), percent recovery rate for methanol extraction followed by Chromabond HR-X SPE clean-up, calibration equation using 0–1000 ppb standard curve, goodness of fit (*R*^2^), and coefficient of variation (CV) between assays (inter-assay) for each analyte measured in 10–15 mg hair samples. The matrix effect (ME_ionization_) reported in percentage with 100% indicating no matrix effect, less than 100% ionization suppression, and over 100% ionization enhancement.

Category and Class of Analyte	Analyte	Mode ESI	Retention Time (min)	Precursor Ion (*m/z*)	Product Ions (1/2)	Cone (V)	EC (ev)	LOD (pg/mg)	LOQ (pg/mg)	Recovery Rate	Calibration Equation	*R* ^2^	CV	ME_ionization_ (%) (100 ppb)
Systemic herbicide	2,4-dichlorophenoxyacetic acid (2,4-D)	Negative	8.66	218.79	160.80	30	14	14.7	44.6	85.1%	*y* = 899,916*x* + 311.78	1.00	11.7%	83.6%
Systemic herbicide	Atrazine	Positive	9.54	216.04	216.10/173.90	55	18	2.1	6.4	101.5%	*y* = 8962.7*x* + 2265.8	0.999	4.3%	101.1%
Systemic herbicide	3,6-Dichloro-2-methoxybenzoic acid (Dicamba)	Negative	7.08	219.00	175.00/145.00	20	10	17.5	53.1	76.8%	*y* = 177.04*x* + 630.89	0.999	13.2%	69.9%
Systemic herbicide	Glyphosate	Positive	7.37	171.23	125.14/111.02	30	Tune	5.7	17.4	70.8%	*y* = 2967.9ln(x) + 56,967	0.993	9.8%	87.0%
Carbonate insecticide	Carbaryl	Positive	9.34	202.04	144.9/127.09	25	12	0.12	0.36	121.0%	*y* = 14,260*x* + 1502.3	0.999	3.4%	98.9%
Neonicotinoid insecticide	Clothianidin	Positive	7.43	249.96	168.70/132.07	25	12	27.7	84.0	103.3%	*y* = 533.3*x* − 3515.4	0.993	6.9%	96.9%
Neonicotinoid insecticide	Imidacloprid	Positive	7.42	255.96	208.9/175.16	35	16	1.2	4.0	84.3%	*y* = 6 × 10^6^*x* + 2562.3	1.00	1.1%	96.7%
Neonicotinoid insecticide	Thiamethoxam	Positive	6.96	291.96	210.8/181.13	30	12	0.5	1.6	120.5%	*y* = 5138*x* + 12.222	0.999	5.7%	98.2%

**Table 3:** Concentrations of target pesticides in each sample (hair pool) of bat hair from three bats using the optimal extraction and clean-up protocol, methanol extraction and Chromabond HR-X SPE column. ND is not detected. LOQ is limit of quantification.

Pesticide	Audrain Hair Pool (pg/mg)	Platte Hair Pool (pg/mg)	St. Louis Hair Pool (pg/mg)
2,4-D	<LOQ	<LOQ	431.9
Atrazine	83.3	40.5	ND
Carbaryl	41.4	216.7	ND
Clothianidin	1949.8	ND	841.2
Dicamba	1574.8	<LOQ	ND
Glyphosate	3580.8	4505.2	<LOQ
Imidacloprid	10.6	13.57	<LOQ
Thiamethoxam	45.5	46.28	ND

## Data Availability

Data is contained within this manuscript.

## Supplementary Materials

The following are available online at https://www.mdpi.com/article/10.3390/toxics10020073/s1, Figure S1: Big brown bat (*Eptesicus fuscus*) with intrascapular hair clipped in preparation for VHF transmitter placement.
