# Supplementary material for: Development of an LC-MS/MS Method for Non-Invasive Biomonitoring of Neonicotinoid and Systemic Herbicide Pesticide Residues in Bat Hair
Source: Toxics. 2022 Feb 5;10(2):73. doi: 10.3390/toxics10020073 (PMC8878529; doi:10.3390/toxics10020073)
Supplement: Supplementary file 1 [file toxics-10-00073-s001.zip › toxics-1365145-supplementary.pdf]

toxics

MDPI

Supplementary Materials: Development of an LC-MS/MS Method for Non-Invasive Biomonitoring of Neonicotinoid and Systemic Herbicide Pesticide Residues in Bat Hair

Sarah E. Hooper, Sybill K. Amelon and Chung-Ho Lin

Hair is required to be clipped prior to placing a VHF bat transmitter in order to allow the VHF transmitter to be glued to the skin using surgical glue. This hair is normally discarded; however our hypothesis was that we could use this normally discarded hair for pesticide residue analysis. Therefore, the weight of the samples was determined by the weight of the hair that is commonly removed during transmitter placement. The figure below illustrates a big brown bat whose hair has been clipped in preparation for a transmitter.

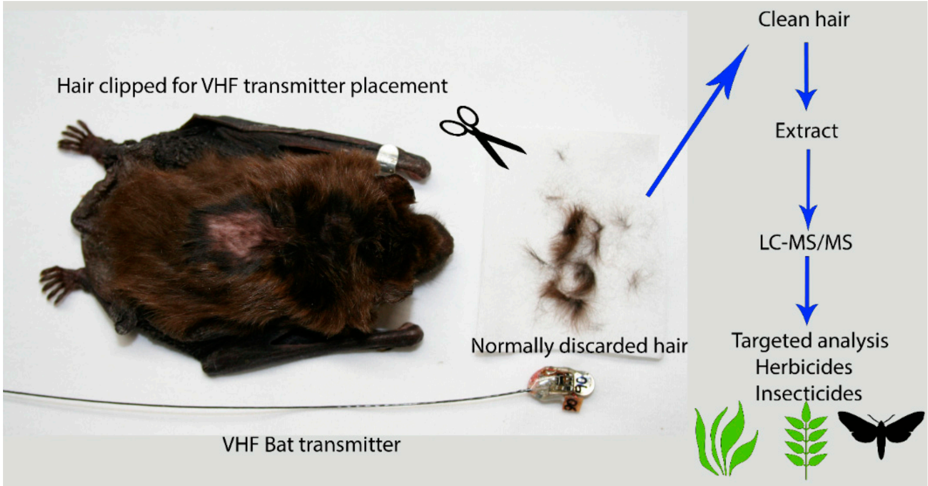

Figure S1. Big brown bat (*Eptesicus fuscus*) with intrascapular hair clipped in preparation for VHF transmitter placement.
